# An Elusive Diagnosis of Castleman Disease

**DOI:** 10.1016/j.atssr.2024.02.018

**Published:** 2024-03-27

**Authors:** Yasmine Rifai, Rohun Bhagat, Sudish Murthy, Alejandro Bribriesco

**Affiliations:** 1 Hackensack Meridian School of Medicine, Nutley, New Jersey; 2 Department of Thoracic & Cardiovascular Surgery, Cleveland Clinic, Cleveland, Ohio; 3 Department of Thoracic & Cardiovascular Surgery, Louis Stokes Cleveland Veterans Affairs Medical Center, Cleveland, Ohio

## Abstract

We present the case of a 41-year-old man with an anterior mediastinal mass and constellation of clinical symptoms, including dyspnea, pleural effusions, pericardial effusions, renal insufficiency, and pancytopenia. After inconclusive results on several laboratory tests and a nondiagnostic surgical biopsy specimen, a specimen from a second surgical biopsy identified the patient’s condition as Castleman disease associated with TAFRO (thrombocytopenia, anasarca, fevers, reticulin myelofibrosis, organomegaly) syndrome. This case highlights the importance of obtaining large tissue biopsy samples, interval follow-up, and acknowledging cognitive biases.

Castleman disease is a rare lymphoproliferative disorder characterized by excessive proliferation of B lymphocytes and plasma cells in lymphoid organs.^1^ Castleman disease can be unifocal or multifocal and characterized into subtypes, including hyaline vascular, plasma cell type, and human herpesvirus-8 associated.^1^ This disorder can mimic both benign and malignant disease, with a myriad of clinical presentations. In the setting of enlarged lymph nodes, the diagnosis involves a set of clinical criteria, with the most important being tissue diagnosis.^1^

Multifocal Castleman disease can also be associated with TAFRO (thrombocytopenia, anasarca, fevers, reticulin myelofibrosis, and organomegaly) syndrome, which is an interleukin-6–driven pathology.^2^ Although TAFRO syndrome shares histopathologic lymph node features with Castleman disease, some sources state this syndrome should be classified as its own clinical entity.^3^ The only reported incidence of TAFRO is 0.9 to 4.9 per million people in Japan.^4^ TAFRO syndrome manifests acutely and is associated with rapid patient deterioration.^2^ One study reported survival of two-thirds of the patient cohort at 24 months from diagnosis.^4^

Diagnostic criteria include thrombocytopenia (<100,000 platelets/mm^3^), anasarca, fever (>37.5 °C) or inflammation (C-reactive protein >2 mg/dL), reticulin myelofibrosis, organomegaly, anemia (hemoglobin <10 g/dL), and renal insufficiency (creatinine >1.2 mg/dL in men, >1.0 mg/dL in women).^4^ Another important criterion is histopathologic findings of lymph nodes; however, due to thrombocytopenia, biopsies can pose significant risk. Autoimmune dysfunction is observed in almost half of patients and presents with anti–Sjögren-syndrome-related antigen A antibodies, rheumatoid-associated antibodies, and platelet-associated antibodies.^5^ TAFRO syndrome is uncommon but has important diagnostic and therapeutic implications for multidisciplinary teams to consider.

The patient, a 41-year-old man, presented in January 2023 with chest pain, tachycardia, and bilateral lower extremity edema. Laboratory results revealed hemoglobin of 10.3 g/dL, platelets of 114,000 per mm^3^, white blood cell count of 15 × 10^9^/L, creatinine of 1 mg/dL, and D-dimer of 4280 mg/dL. Chest computed tomography (CT) showed an anterior mediastinal mass measuring 7.5 cm with thoracic lymphadenopathy and a large pericardial effusion. Positron emission tomography (PET) scan (Figure 1) demonstrated increased [^1^⁸F]fluorodeoxyglucose avidity (standardized uptake value, 4.0). The sample from a CT-guided core needle biopsy yielded inconclusive results. Given concern for malignancy, thoracic surgery was consulted for a surgical biopsy and opted for a left video-assisted thoracoscopic surgery (VATS) approach.

**Figure 1:**
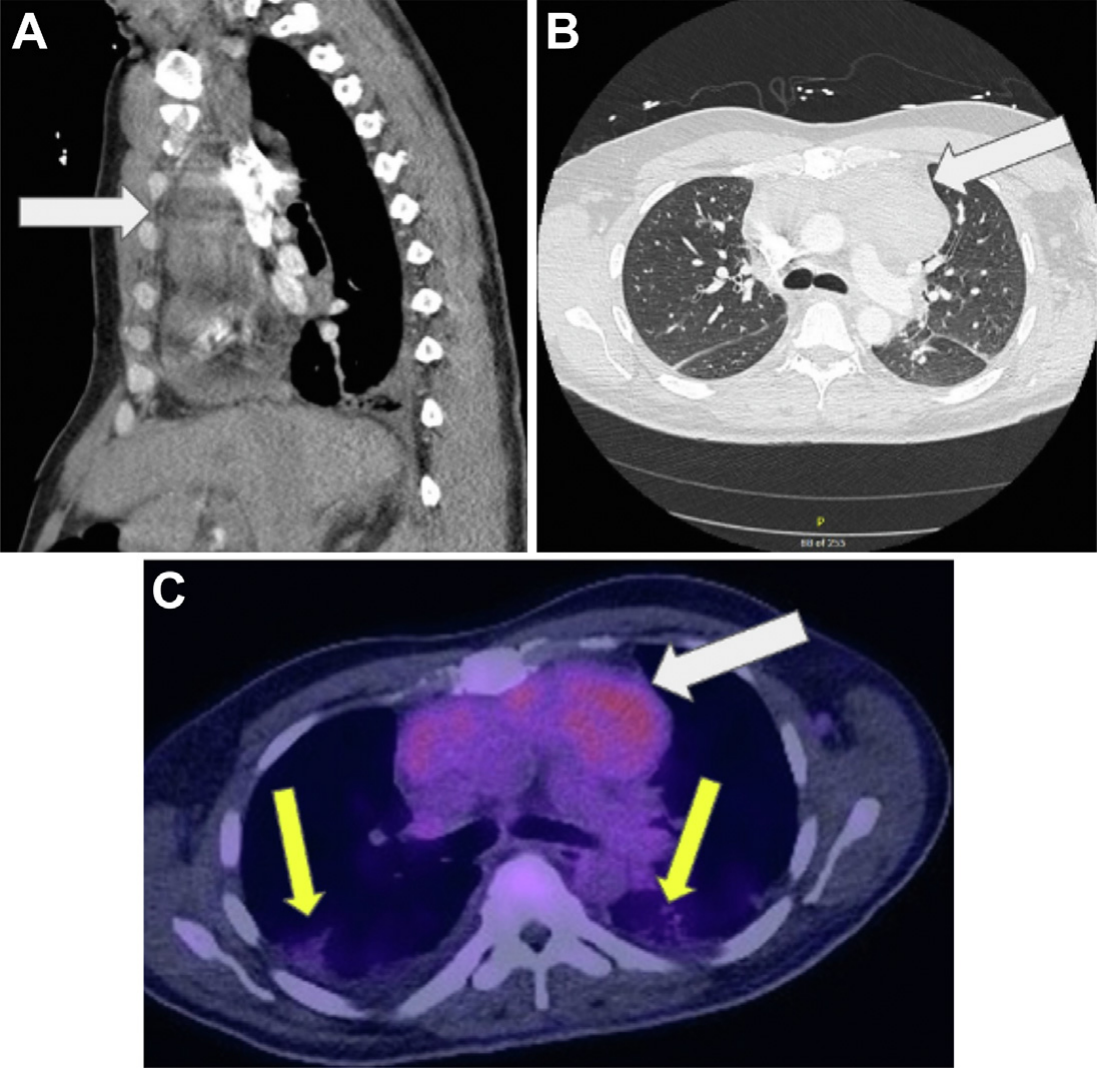
(A) Sagittal chest computed tomography (CT). (B) Axial chest CT. (C) Axial chest CT/positron emission tomography scan. The white arrows point to the anterior mediastinal mass, and the yellow arrows point to pleural effusion.

The mass was visualized and adherent to lung, thymic tissue, and pericardium. En bloc biopsy of the mass with wedge resection of the involved left upper lobe and pericardium was performed (Figure 2). Before the pathology report was finalized, the patient convalesced and met criteria for discharge on postoperative day 4, with scheduled outpatient follow-up. Ultimately, the pathology report was noncontributory and reported as benign fragments of thymic epithelia and chronic inflammation, with no evidence of malignancy.

**Figure 2:**
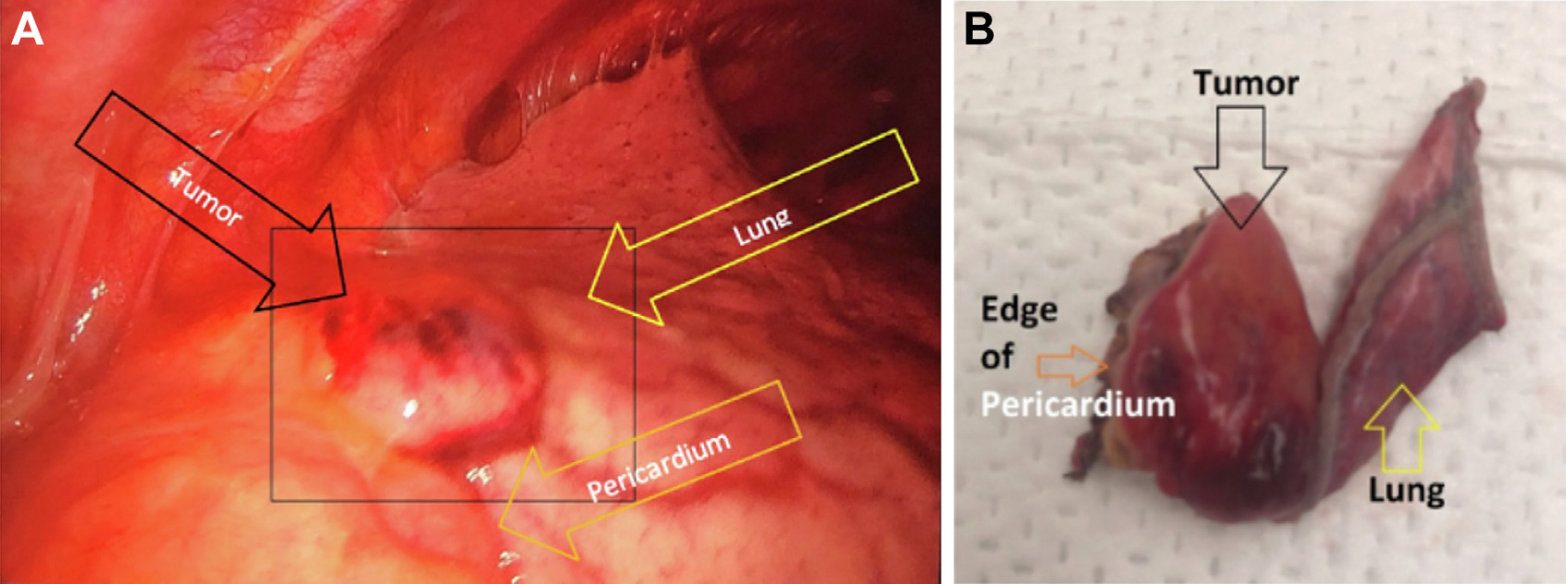
(A) Mediastinal mass with left upper lobe of the lung laterally and superiorly and pericardium inferiorly. (B) Gross specimen of mediastinal mass consisting of lung and pericardium in peripheral margins.

Despite attempts to reach the patient, he was lost to follow-up for 6 months, when he presented to the emergency department with worsening symptoms, including tachycardia, dyspnea, and bilateral lower-extremity edema. Laboratory results revealed worsened pancytopenia (hemoglobin, 3.9 g/dL; 11,000 platelets/mm^3^, white blood cells, 1.82 × 10^9^/L), acute renal failure (creatinine, 2 mg/dL; blood urea nitrogen, 84 mg/dL) and inflammation (C-reactive protein, 4.8 mg/dL). Chest CT (Figure 3) demonstrated bilateral pleural effusions, pericardial effusion, and interval growth of the mediastinal mass.

**Figure 3:**
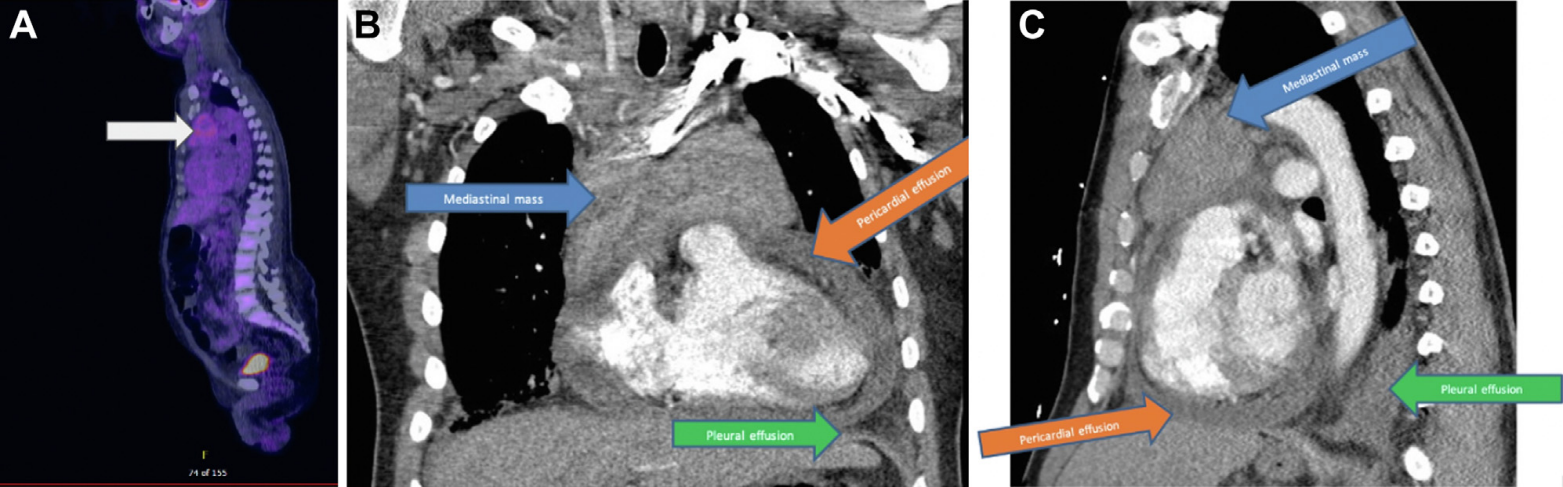
(A) Sagittal chest computed tomography (CT). (B) Coronal chest CT revealing mediastinal mass (blue arrow), pericardial effusion (orange arrow), and pleural effusion (green arrow). (C) Sagittal chest CT.

Owing to the patient’s pancytopenia, he was transferred to the medical intensive care unit. A bone marrow biopsy sample revealed hypercellular marrow due to panhyperplasia. Autoimmune antibodies, including anti–Sjögren-syndrome-related antigen A and antinuclear antibody, were positive, without symptoms of Sjögren disease. Repeat PET/CT scan (Figure 3) redemonstrated the mediastinal mass, with a standardized uptake value of 3.5, and lymph nodes throughout the body with variable hypermetabolism relative to his prior scan. After multidisciplinary discussion, definitive tissue diagnosis of the anterior mediastinal mass was recommended to guide treatment. The patient received intravenous immunoglobulin and platelet and red blood cell transfusions for resuscitation and preparation for surgery. On day 10 of his hospital stay, he underwent a left anterior mediastinotomy (Chamberlain procedure).

During the procedure, a scalpel was used to obtain multiple large incisional biopsy specimens, including samples from deep within the mass. These samples revealed hyperplastic thymic tissue with plasmacytosis, consistent with Castleman disease. With the diagnosis established, the clinical presentation was identified as TAFRO syndrome. The patient recovered uneventfully from surgery and was initiated on anti–interleukin-6 therapy (siltuximab). After a hospital course complicated by manifestations of his TAFRO syndrome, the patient was ultimately discharged home on postoperative day 29. He maintains regular follow-up with the clinic, with his most recent office visit with pulmonology in December 2023.

## Comment

This case raises 2 distinct issues. The first is identifying a rare disease with an uncommon presentation. A common differential when evaluating an anterior mediastinal mass includes lymphomas, thymomas, teratomas, and other germ cell tumors.^6^ A definitive diagnosis is obtained by biopsy and histologic examination.^7^ Methods of biopsy include transbronchial or CT-guided transthoracic core-needle biopsy, mediastinoscopy, VATS, and open surgical biopsy.^7^ In our case, the patient underwent nondiagnostic CT-guided core-needle biopsy and noncontributory VATS, despite a seemingly generous en bloc tissue specimen.

Over time, practitioners and trainees frequently develop availability heuristics that lead to cognitive biases. In hindsight, the patient’s initial presentation of pancytopenia, anasarca, organomegaly, fever, and a mediastinal mass did meet the criteria for Castleman-TAFRO disease. However, heuristics inform us, based on the patient’s age, presentation, and prevalence of diagnosis, that lymphoma is most likely. Additionally, although Castleman disease is well known, it is likely that practitioners are not as familiar with the TAFRO syndrome subset, such that a unifying diagnosis may be elusive despite involvement of a multidisciplinary team. This further underscores the importance of a tissue diagnosis. In the future, proceeding directly to the Chamberlin procedure in the setting of a complex clinical situation will be the preferred approach.

The second issue is, unfortunately, too common in health care—loss to follow-up. As medical practitioners, it is imperative to educate patients on their condition and the importance of follow-up after discharge. It is also vital to discuss patients’ social determinants of health and barriers that impact their ability to understand their condition or attend outpatient visits. Had the team remained in contact with the patient, there may have been opportunity for timely reevaluation of symptoms, laboratory analysis, and consideration of additional diagnostics to establish a diagnosis and initiate treatment.

Castleman disease associated with TAFRO is a rare, severe condition that is vexing for clinicians and patients. Despite the increased risk of biopsy, the diagnosis requires histologic evidence to solidify the syndrome of thrombocytopenia, anasarca, fevers, reticulin myelofibrosis, and organomegaly as TAFRO. Multidisciplinary teams, including surgeons, should counsel patients throughout to facilitate optimal outcomes.
